# Etiological Factors of Orofacial Clefts in a Hospital-Based Population: A Descriptive Analytical Study

**DOI:** 10.7759/cureus.98868

**Published:** 2025-12-10

**Authors:** Mohammad Naffizuddin, Chaganti Ashok, Sowjanya Gunukula, Rvbs Sarma, Bhavana Sujanamulk, Bharani Krishna Takkella, Chukka Ram Sunil, Varri Sujana, Ashwini Kumar

**Affiliations:** 1 Oral and Maxillofacial Surgery, Care Dental College and Hospital, Guntur, IND; 2 Conservative Dentistry and Endodontics, Nimra Institute of Dental Sciences, Vijayawada, IND; 3 Healthcare Administration, Western Kentucky University, Bowling Green, USA; 4 Dentistry, University at Buffalo, Buffalo, USA; 5 General Dentistry, Flomo Dental Clinic, Flower Mound, USA; 6 Physiology, Dr. Pinnamaneni Siddhartha Institute of Medical Sciences and Research Foundation, Vijayawada, IND; 7 Oral and Maxillofacial Surgery/Diagnostic Sciences, Division of Oral Medicine and Radiology, Faculty of Dentistry, Najran University, Najran, SAU; 8 Oral Medicine and Radiology, Drs. Sudha and Nageswara Rao Siddhartha Institute of Dental Sciences, Vijayawada, IND; 9 Conservative Dentistry and Endodontics, Sibar Institute of Dental Sciences, Guntur, IND; 10 Conservative Dentistry and Endodontics, Himachal Dental College, Sunder Nagar, IND

**Keywords:** cleft lip and palate (clp), cleft lip (cl), cleft palate (cp), consanguinity, folic acid, orofacial clefts (ofc), prenatal infections, prenatal scans, socioeconomic status

## Abstract

Introduction: Cleft lip (CL) and cleft palate (CP) are the most common congenital anomalies affecting the craniofacial region. These conditions occur when the tissues forming the upper lip and the roof of the oral cavity fail to fuse properly during embryonic development, usually in the first trimester. Their etiology is multifactorial. Functionally, CL, CP, and cleft lip and palate (CLP) can lead to significant challenges, including feeding difficulties, speech and hearing problems, and psychological issues in children.

Materials and methods: This study was conducted at Drs. Sudha & Nageswara Rao Siddhartha Institute of Dental Sciences, Chinoutpalli, Andhra Pradesh, India. A total of 404 patients from 2017 to 2025 were evaluated for variables such as age, sex, laterality of CL (complete and incomplete), consanguinity, paternal and maternal age, birth order, prenatal folic acid intake, prenatal maternal infections, family history, previous abortions, abnormalities detected in antenatal scans, and socioeconomic status, using a structured questionnaire. Cleft patterns were classified according to the Kernahan and Stark classification for CL and CP.

Results: Etiological factors, such as male gender, consanguinity, maternal age, prenatal folic acid supplementation history, prenatal infections, abnormalities detected during antenatal ultrasound, and socioeconomic status, were found to be significant for CL, CP, and CLP (p ≤ 0.001). Complete CL occurring on the left side, paternal age, first-born status, and family history were also statistically significant (p = 0.01). In contrast, incomplete CL (p = 0.17), CL with alveolar involvement (p = 0.23), and previous abortion history (p = 0.093) showed no significant association. Multivariate logistic regression was used to determine odds ratios (ORs) and assess confounding factors. Consanguinity, prenatal maternal folic acid deficiency, maternal age between 30-35 and 35-40 years, paternal age between 35 and 40 years, first-born status, and lower socioeconomic status were identified as the strongest risk factors for CL, CP, and CLP. Prenatal infections were more strongly associated with CL, while positive family history was more likely associated with CP. Abnormalities detected during prenatal scans were associated with CP and CLP.

Conclusion: The present study identified several significant risk factors for CL, CP, and CLP. The prevalence was higher among male children. Incomplete CL and CL with alveolar involvement showed no association with gender. Key contributing factors included consanguineous marriages, advanced maternal and paternal age at conception, folic acid deficiency, first-born status, and lower socioeconomic status. Additional associated factors were prenatal infections (for CL), positive family history (for CP), and abnormalities detected during prenatal scans (for CP and CLP). Previous abortion history showed no association with clefts.

## Introduction

Cleft lip (CL), cleft palate (CP), and cleft lip and palate (CLP) are among the most frequently occurring congenital abnormalities affecting the head and neck region. Together, they account for about 65% of all anomalies in this anatomical area. Globally, these defects affect around 5% of all newborns [1]. According to the World Health Organization, a child is born with a cleft every two minutes. In India (the second most populous country, with over 1 billion people), approximately 28,600 infants are born every year with CL, CP, or CLP. Orofacial clefts (OFCs), which mainly involve the upper lip and palate, occur in about 1 in every 700 births [2], although the incidence varies across ethnic groups. These malformations arise during early embryonic development, beginning around the fourth week of gestation. At this stage, facial features form through the interaction of the branchial (pharyngeal) arches. Som and Naidich described that the upper lip and jaw develop from the fusion of the medial-nasal and maxillary processes, while the lower jaw forms from the mandibular process. The philtrum, the vertical groove between the nose and upper lip, is created when the two medial-nasal processes merge [3].

Palatal development begins around the sixth week of embryogenesis. The primary palate forms from a cellular mass between the maxillary surfaces, while the secondary palate includes the remaining hard and soft palatal structures. Any disruption or irregularity in these fusion processes can result in clefts [3]. A complete cleft indicates total failure of fusion, while partial or incomplete clefts indicate partial merging. The global prevalence of OFCs is approximately 1 in 700 live births, making up nearly half of all craniofacial anomalies [4]. According to Allam et al., the birth prevalence of CL with or without CP ranges from 3.4 to 22.9 per 10,000 births, while CP alone ranges from 1.3 to 25.3 per 10,000 births [5]. Ethnicity influences incidence: Asian populations show the highest rates (0.82-4.04 per 1000 live births), Caucasians show intermediate rates (0.9-2.69 per 1000), and Africans have the lowest rates (0.18-1.67 per 1000) [6].

Jamilian et al. noted that children born with clefts often require long-term, multidisciplinary care that may extend into adulthood. They face higher rates of mortality and morbidity than unaffected individuals, along with significant medical, psychological, social, and financial burdens [7]. Management may include maxillofacial surgery, psychological support, and orthodontic treatment. Because clefts are complex conditions, their risk factors are also multifactorial. They are believed to result from a combination of genetic predisposition and environmental influences [8]. Lei et al. reported that although clefts tend to appear in families, inheritance patterns are not clearly defined, making risk prediction difficult [9]. In consanguineous marriages, shared parental genetics increase the chance of inheriting defective genes, which elevates the risk of clefts in offspring [10].

The current study was a descriptive analytical study aimed at determining the prevalence and demographic patterns of CL, CP, and CLP. It also assessed parental and prenatal confounding risk factors such as age, sex, laterality, consanguinity, maternal and paternal age, birth order, folic acid use, maternal infections, family history, abortion history, prenatal detection of anomalies, and socioeconomic status, and evaluated their association with CL, CP, and CLP.

## Materials and methods

Study design and setting

The current study was a descriptive, hospital-based, cross-sectional analytical study. Written informed consent was obtained from the parents or legal guardians of all patients prior to recruitment. The study was conducted in accordance with the ethical standards of the Declaration of Helsinki (2013 revision).

Study population and sample size

The study population consisted of 404 patients (243 males and 161 females) presenting with CL, CP, or CLP. The study protocol was submitted to and approved by the Institutional Ethics Committee of Drs. Sudha & Nageswara Rao Siddhartha Institute of Dental Sciences (IEC No. 27/2017). Participants were recruited through stratified convenience sampling from all outpatients attending the Department of Oral Medicine and Radiology at Drs. Sudha & Nageswara Rao Siddhartha Institute of Dental Sciences, Chinoutpalli, Andhra Pradesh, India, over an eight-year period from January 2017 to March 2025. Parents or legal guardians were interviewed using a structured questionnaire developed for the study (Supplemental material 1). Pilot testing was conducted on the first 10 cases to ensure clarity and reliability

Inclusion criteria

The diagnosis of CL, CP, or CLP was confirmed through clinical examination by oral physicians with more than three years of experience. Parents or legal guardians provided consent for participation in the study and for surgical intervention at our center.

Exclusion criteria

Patients were excluded if their parents or guardians declined participation for unspecified reasons, if they were referred for a second opinion or counseling but were unwilling to undergo surgical intervention at the institute, or if they had incomplete records or missing parental information regarding prenatal or postnatal factors. Additionally, questionnaires that were not in agreement between the data analysts were excluded from the study.

Data collection tools and techniques for patient recruitment

A structured case record proforma was used to standardize data collection. The questionnaire administered to parents was provided in the native language (Telugu). Each patient was assigned a unique number linked to their outpatient ID, and a separate case chart was created in the institute’s data monitoring system for easy access. In this study, data were collected prospectively in real time by two data analysts independently; only cases where both analysts agreed were included. Previous medical and prenatal hospital records were thoroughly reviewed to minimize subjective recall errors. Demographic information such as age, gender, and socioeconomic status was cross-checked and verified using the Aadhar card (a unique identification number issued in India). Patients were also informed about the possibility of recall bias and its impact on the study, in order to encourage accurate responses. Socioeconomic status was classified using the Kuppuswamy scale [11].

The questionnaire consisted of the following sections: (1) demographic characteristics: age, gender, and socioeconomic status; (2) clinical characteristics of cleft anomalies: complete CL and gender, incomplete CL and gender, and distribution of CL with alveolar involvement; and (3) parental and prenatal factors: consanguinity (defined as marriage between biological relatives up to second cousins), maternal and paternal age at conception, birth order of the affected child, periconceptional folic acid supplementation, history of febrile prenatal illness, family history of OFC, previous abortions, prenatal ultrasound findings, and abnormalities detected on scans.

Cleft patterns were classified according to the Kernahan and Stark classification [12] (Table 1).

**Table 1:** Kernahan and Stark classification of CL and CP CL: cleft lip, CP: cleft palate.

Number	Anatomical location	Clinical significance
Block 1	Right nasal floor	Present/absent
Block 2	Left nasal floor	Present/absent
Block 3	Right lip	Present/absent
Block 4	Left lip	Present/absent
Block 5	Right alveolus	Present/absent
Block 6	Left alveolus	Present/absent
Block 7	Right hard palate anterior to the incisive foramina	Present/absent
Block 8	Left hard palate anterior to the incisive foramina	Present/absent
Blocks 9 and 10	Hard palate posterior to the incisive foramina	Present/absent
Block 11	Soft palate	Present/Absent

Statistical analysis

Data were compiled using Microsoft Excel (Microsoft Corp., Redmond, WA, USA), and statistical analysis was performed using IBM SPSS Statistics for Windows, Version 24.0 (Released 2016; IBM Corp., Armonk, NY, USA). Descriptive statistics were used to summarize the data. Normality of the variables was assessed, and the chi-square test was used to evaluate associations between categorical variables, with specific values assigned to each parameter. The Shapiro-Wilk test was performed to assess statistical significance. The level of significance was set at 5%, with p ≤ 0.001 considered highly significant, p ≤ 0.05 significant, and p > 0.05 insignificant. Subsequently, the data were processed for multivariable analysis using multiple logistic regression with likelihood ratio tests to determine the impact of independent variables on cleft formation. The β coefficient was evaluated to indicate how a one-unit change in an independent variable affects the log odds of the dependent variable. Standard error was measured to determine the accuracy of sample statistics in representing the true population parameter. Degrees of freedom (df) reflected the number of independent variables free to vary in the analysis. Odds ratio (OR), interpreted as the exponential of the β value, was calculated to determine the strength of association between the exposure and outcome, where a higher OR indicates a greater likelihood of the outcome. The 95% confidence interval (CI) represented the range of plausible values across repeated samples.

## Results

The numerical values for each parameter were considered, and the analysis was performed accordingly. In Table 2, the numerical values representing the number of cases and their corresponding percentages were clarified and presented as mean ± SD. Consanguinity, advanced maternal age, lack of prenatal folic acid supplementation, prenatal infections, abnormalities detected during prenatal ultrasound, and lower socioeconomic status were identified as clear risk factors for CL, CP, and CLP, with high statistical significance (p ≤ 0.001) (Tables 3-15). Complete CL, particularly with left-sided involvement, was more common in males. High paternal age, birth order, and a positive family history also showed statistical significance (p = 0.01). In contrast, incomplete CL laterality did not show a significant association with gender (p = 0.17), and alveolar involvement did not demonstrate a significant correlation with gender (p = 0.23). Additionally, a previous history of abortion did not show a statistically significant association with the occurrence of CL, CP, or CLP (p = 0.093).

**Table 2:** Age distribution of subjects based on gender (N = 404)

Gender	Sample size	Mean ± SD
Male	243	121.11 ± 92.56
Female	161	108.59 ± 89.68

**Table 3:** Distribution of cleft deformities by gender In 404 patients, 121 had CL (68 (56.2%) males, 53 (43.8%) females), 104 had CP (44 (42.3%) males, 60 (57.7%) females), and 179 had CLP (131 (73.3%) males, 48 (26.8%) females). The CLP pattern was more commonly observed in males, with a chi-square value of 27.288 and a p-value < 0.001, indicating high statistical significance. CL: cleft lip, CP: cleft palate, CLP: cleft lip and palate.

Gender	CL, n (%)	CP, n (%)	CLP, n (%)	Total, n (%)	Chi-square value	p-value
Male	68 (56.2)	44 (42.3)	131 (73.2)	243 (60.1)	27.288	<0.001
Female	53 (43.8)	60 (57.7)	48 (26.8)	161 (39.9)		
Total	121 (100)	104 (100)	179 (100)	404 (100)		

**Table 4:** Distribution of complete CL (laterality) by gender In 98 patients with complete CL, 56 (57.1%) were males, of whom 4 had right-sided clefts, 32 had left-sided clefts, and 20 had bilateral clefts. Among the 42 (42.9%) females with complete CL, 10 had right-sided clefts, 24 had left-sided clefts, and 8 had bilateral clefts. This finding demonstrates that left-sided clefts are more common than right-sided clefts in both genders, and bilateral clefts were more frequent in males. The chi-square value was 23.73, with a statistically significant p-value of 0.01. CL: cleft lip.

Gender	Complete CL			Total, n (%)	Chi-square value	p-value
	Right, n (%)	Left, n (%)	Bilateral, n (%)		23.73	0.01
Males	4 (28.6)	32 (57.1)	20 (71.4)	56 (57.1)		
Females	10 (71.4)	24 (42.9)	8 (28.6)	42 (42.9)		
Total	14 (100.0)	56 (100.0)	28 (100.0)	98 (100.0)		

**Table 5:** Distribution of incomplete CL (laterality) by gender In 23 patients with incomplete CL, 12 (52.2%) were males, of whom 6 had right-sided clefts and 6 had bilateral clefts. Among the 11 (47.8%) females with incomplete CL, 5 had right-side clefts and 6 had left-side clefts. This finding shows that left-sided clefts were more common in females, while bilateral clefts were more frequent in males. However, these differences were not statistically significant, with a chi-square value of 17.54 and a p-value of 0.17. CL: cleft lip.

Gender	Incomplete CL			Total, n (%)	Chi-square value	p-value
	Right, n (%)	Left, n (%)	Bilateral, n (%)		17.54	0.17
Males	6 (54.5)	0 (0.0)	6 (100.0)	12 (52.2)		
Females	5 (45.5)	6 (100.0)	0 (0.0)	11 (47.8)		
Total	11 (100.0)	6 (100.0)	6 (100.0)	23 (100.0)		

**Table 6:** Distribution of CL with alveolar involvement by gender In 27 patients with CL involving the alveolus, 17 (63%) were males, of whom 5 (55.6%) had right-sided clefts, 10 (66.7%) had left-sided clefts, and 2 (66.7%) had bilateral clefts. Among the 10 (37%) females with CL involving the alveolus, 4 (44.4%) had right-sided clefts, 5 (33.3%) had left-sided clefts, and 1 (33.3%) had bilateral cleft. This finding shows that left-sided CL with alveolar involvement was more common in both genders; however, the association was not statistically significant (chi-square = 19.55, p = 0.23). CL: cleft lip.

Gender	CL with involvement of the alveolus			Total, n (%)	Chi-square value	p-value
	Right, n (%)	Left, n (%)	Bilateral, n (%)		19.55	0.23
Males	5 (55.6)	10 (66.7)	2 (66.7)	17 (63.0)		
Females	4 (44.4)	5 (33.3)	1 (33.3)	10 (37.0)		
Total	9 (100.0)	15 (100.0)	3 (100.0)	27 (100.0)		

**Table 7:** Association between the type of cleft and consanguinity In the current study, 273 parents of children with clefts reported a history of consanguineous marriage. Among them, 85 (70.2%) had children with cleft lip, 82 (78.8%) with cleft palate, and 106 (59.2%) with cleft lip and palate. The chi-square value was 12.13 with a p-value ≤ 0.001, indicating a highly significant association.

Consanguinity	Diagnosis			Total n (%)	Chi-square value	p-value
	CL, n (%)	CP, n (%)	CLP, n (%)			
Consanguineous	85 (70.2)	82 (78.8)	106 (59.2)	273 (67.6)	12.13	≤0.001
Non-consanguineous	36 (29.8)	22 (21.2)	73 (40.8)	131 (32.4)		
Total	121 (100.0)	104 (100.0)	179 (100.0)	404 (100.0)		

**Table 8:** Association between the type of cleft and paternal age Among the 121 CL cases, 104 CP cases, and 179 CLP cases, paternal age was distributed as follows: 20-25 years, 33 (8.2%); 26-30 years, 45 (11.1%); 31-35 years, 89 (22%); and 36-40 years, 237 (58.7%). The association was statistically significant, with a chi-square value of 16.878 and p = 0.01. CL: cleft lip, CP: cleft palate, CLP: cleft lip and palate.

Paternal age	Diagnosis			Total, n (%)	Chi-square value	p-value
	CL, n (%)	CP, n (%)	CLP, n (%)			
20-25	10 (8.3)	5 (4.8)	18 (10.1)	33 (8.2)	16.878	0.01
26-30	24 (19.8)	9 (8.7)	12 (6.7)	45 (11.1)		
31-35	22 (18.2)	28 (26.9)	39 (21.8)	89 (22.0)		
36-40	65 (53.7)	62 (59.6)	110 (61.5)	237 (58.7)		
Total	121 (100.0)	104 (100.0)	179 (100.0)	404 (100.0)		

**Table 9:** Association between the type of cleft and maternal age Among the 121 CL cases, 104 CP cases, and 179 CLP cases, maternal age was distributed as follows: 20-25 years, 32 (7.9%); 26-30 years, 51 (12.6%); 31-35 years, 224 (55.4%); and 36-40 years, 97 (24%). The association was highly significant, with a chi-square value of 19.44 and p ≤ 0.001. CL: cleft lip, CP: cleft palate, CLP: cleft lip and palate.

Maternal age	Diagnosis			Total, n (%)	Chi-square value	p-value
	CL, n (%)	CP, n (%)	CLP, n (%)			
20-25	11 (9.1)	6 (5.8)	15 (8.4)	32 (7.9)	19.444	≤0.001
26-30	13 (10.7)	8 (7.7)	30 (16.8)	51 (12.6)		
31-35	73 (60.3)	50 (48.1)	101 (56.4)	224 (55.4)		
36-40	24 (19.8)	40 (38.5)	33 (18.4)	97 (24.0)		
Total	121 (100.0)	104 (100.0)	179 (100.0)	404 (100.0)		

**Table 10:** Association between the type of cleft and order of birth Among 404 children, 283 (70%) were first-born, 92 (22.8%) were second-born, 25 (6.2%) were third-born, and 4 (1%) were fourth-born. The association was statistically significant, with a chi-square value of 19.455 and p = 0.01. CL: cleft lip, CP: cleft palate, CLP: cleft lip and palate.

Order of child birth	CL, n (%)	CP, n (%)	CLP, n (%)	Total, n (%)	Chi-square value	p-value
First	88 (72.7)	74 (71.2)	121 (67.6)	283 (70.0)	19.455	0.01
Second	16 (13.2)	25 (24.0)	51 (28.5)	92 (22.8)		
Third	15 (12.4)	4 (3.8)	6 (3.4)	25 (6.2)		
Fourth	2 (1.7)	1 (1.0)	1 (0.6)	4 (1.0)		
Fifth	0 (0.0)	0 (0.0)	0 (0.0)	0 (0.0)		
Total	121 (100)	104 (100)	179 (100)	404 (100)		

**Table 11:** Association between the type of cleft and prenatal folic acid supplementation Among the 404 children, 62 (15.3%) had a maternal history of folic acid supplementation, while 342 (84.7%) did not. The association was highly significant, with a chi-square value of 8.105 and p < 0.001. CL: cleft lip, CP: cleft palate, CLP: cleft lip and palate.

Prenatal folic acid supplementation	CL, n (%)	CP, n (%)	CLP, n (%)	Total, n (%)	Chi-square value	p-value
Yes	28 (23.1)	12 (11.5)	22 (12.3)	62 (15.3)	8.105	≤0.001
No	93 (76.9)	92 (88.5)	157 (87.7)	342 (84.7)		
Total	121 (100)	104 (100)	179 (100)	404 (100)		

**Table 12:** Association between the type of cleft and prenatal infections Out of 404 mothers, 346 (85.6%) had a history of prenatal infections, while 58 (14.4%) did not. The association was highly significant, with a chi-square value of 9.136 and p ≤ 0.001. CL: cleft lip, CP: cleft palate, CLP: cleft lip and palate.

Prenatal infections	Diagnosis			Total, n (%)	Chi-square value	p-value
	CL, n (%)	CP, n (%)	CLP, n (%)			
Yes	94 (77.7)	94 (90.4)	158 (88.3)	346 (85.6)	9.136	≤0.001
No	27(22.3)	10 (9.6)	21 (11.7)	58 (14.4)		
Total	121 (100.0)	104 (100.0)	179 (100.0)	404 (100.0)		

**Table 13:** Association between the type of cleft and family history Among 404 patients, 300 (74.3%) had a positive family history, while 104 (25.7%) had no family history. The association was statistically significant, with a chi-square value of 8.450 and p = 0.01. CL: cleft lip, CP: cleft palate, CLP: cleft lip and palate.

Family history	CL, n (%)	CP, n (%)	CLP, n (%)	Total, n (%)	Chi-square value	p-value
Yes	101 (83.5)	70 (67.3)	129 (72.1)	300 (74.3)	8.450	0.01
No	20 (16.5)	34 (32.7)	50 (27.9)	104 (25.7)		
Total	121 (100)	104 (100)	179 (100)	404 (100)		

**Table 14:** Association between the type of cleft and previous history of abortion Among 404 mothers of children with clefts, 100 had a previous history of abortion, while 304 (75.2%) had no such history. The association was not statistically significant, with a chi-square value of 7.875 and p = 0.09. CL: cleft lip, CP: cleft palate, CLP: cleft lip and palate.

Abortion history	Diagnosis			Total, n (%)	Chi-square value	p-value
	CL, n (%)	CP, n (%)	CLP, n (%)			
Positive history of abortion	41 (33.9)	23 (22.1)	36 (20.1)	100 (24.8)	7.875	0.093
Negative history of abortion	80 (66.1)	81 (77.9)	143 (79.9)	304 (75.2)		
Total	121 (100.0)	104 (100.0)	179 (100.0)	404 (100.0)		

**Table 15:** Association between the type of cleft and any abnormalities found in prenatal scans Among 404 patients, 265 (65.5%) had abnormalities detected in prenatal scans, while 139 (34.4%) had no such abnormalities. The association was highly significant, with a chi-square value of 58.535 and p ≤ 0.001. CL: cleft lip, CP: cleft palate, CLP: cleft lip and palate.

Abnormalities detected in prenatal scans	CL, n (%)	CP, n (%)	CLP, n (%)	Total, n (%)	Chi-square value	p-value
Detected	89 (71.1)	52 (55.9)	124 (66.6)	265 (65.5)	58.535	≤0.001
Not detected	36 (28.8)	41 (44.0)	62 (33.3)	139 (34.4)		
Total	125 (100)	93 (100)	186 (100)	404 (100)		

**Table 16:** Association between the type of cleft and socioeconomic status Regarding socioeconomic status, the lower class was most affected, with 296 (73.3%) cases, followed by the lower middle class (48, 11.9%), middle class (36, 8.9%), upper middle class (15, 3.7%), and upper class (9, 2.2%). The association was highly significant, with a chi-square value of 18.991 and p ≤ 0.001. CL: cleft lip, CP: cleft palate, CLP: cleft lip and palate.

Socioeconomic status	CL, n (%)	CP, n (%)	CLP, n (%)	Total, n (%)	Chi-square value	p-value
Lower	89 (73.6)	61 (58.7)	146 (81.6)	296 (73.3)	18.991	≤0.001
Lower middle	13 (10.7)	20 (19.2)	15 (8.4)	48 (11.9)		
Middle	10 (8.3)	14 (13.5)	12 (6.7)	36 (8.9)		
Upper middle	5 (4.1)	6 (5.8)	4 (2.2)	15 (3.7)		
Upper	4 (3.3)	3 (2.9)	2 (1.1)	9 (2.2)		
Total	121 (100)	104 (100)	179 (100)	404 (100)		

Using multivariate logistic regression with likelihood ratio tests, several factors were identified as strongly associated with cleft formation (Tables 17-19). For CL, the highest risk was observed with consanguinity (OR = 2.113), followed by prenatal infections (OR = 2.046), lack of prenatal folic acid supplementation (OR = 1.89), paternal age 35-40 years (OR = 1.89), lower socioeconomic status (OR = 1.83), first-born status (OR = 1.298), maternal age 35-40 years (OR = 1.163), and maternal age 30-35 years (OR = 1.107). For CP, the strongest associations were with consanguinity (OR = 2.39), first-born status (OR = 2.29), lower socioeconomic status (OR = 1.98), paternal age 35-40 years (OR = 1.89), abnormalities detected in fetal anomaly scans (OR = 1.83), maternal age 30-35 years (OR = 1.345), positive family history (OR = 1.23), maternal age 35-40 years (OR = 1.017), and lack of maternal folic acid supplementation (OR = 1.00). For CLP, the highest risks were associated with lower socioeconomic status (OR = 2.79), abnormalities detected during prenatal scans (OR = 2.18), maternal age 30-35 years (OR = 2.15), paternal age 35-40 years (OR = 1.89), consanguinity (OR = 1.64), lack of prenatal folic acid supplementation (OR = 1.31), first-born status (OR = 1.29), and maternal age 35-40 years (OR = 1.19).

**Table 17:** Multivariate logistic regression analysis for CL Using logistic regression, the odds ratio (exponential β) values were calculated to determine the risk of association for cleft lip (CL), with the known intercept at the 95% CI upper and lower bounds. Parents with consanguinity had a 2.113-fold higher risk of having a child with CL, with a 95% CI values ranging from 1.272 to 2.714. Lack of prenatal folic acid supplementation showed a 1.899-fold risk, with a 95% CI values of 1.002-2.132. Prenatal infections were associated with a 2.046-fold increased risk, with a 95% CI bounds of 1.065-2.95. Family history showed a risk of 0.975, with upper and lower 95% CI bounds of 1.234-1.372. Previous abortion history showed a risk of 0.233, with a 95% CI values of 0.135-0.577. Abnormalities detected in prenatal scans showed a risk of 0.618, with a 95% CI values of 0.294-1.0 for CL. Maternal age groups of 20-25, 25-30, 30-35, and 35-40 years showed risks of 0.0512, 0.384, 1.107, and 1.163, with corresponding 95% CI bounds of 0.0216-0.075, 0.190-0.566, 0.016-1.78, and 0.070-2.67. Similarly, paternal age groups showed risks of 0.028, 0.236, 0.821, and 1.893, with a 95% CI bounds of 0.014-0.089, 0.316-0.558, 0.101-1.19, and 0.723-2.189, respectively. Birth-order risks for 1st, 2nd, 3rd, 4th, and 5th children were 1.298, 0.791, 0.784, 0.045, and 0.067, with corresponding 95% CI bounds of 0.118-1.546, 0.438-0.996, 0.390-1.08, 0.225-0.543, and 0.023-0.441. Socioeconomic status also showed varying risks: lower class (OR = 1.836; 95% CI: 1.679-2.57), lower-middle class (OR = 0.975; 95% CI: 0.577-1.324), middle class (OR = 0.837; 95% CI: 0.370-1.99), upper-middle class (OR = 0.567; 95% CI: 0.723-0.999), and upper class (OR = 0.0747; 95% CI: 0.021-0.222) for CL.

Intercept	β	Std. error	df	Odds ratio	95% confidence interval for odds ratio
Consanguinity	0.759	0.276	1	2.113	2.714-1.272
Prenatal folic acid usage	0.641	0.326	1	1.899	2.132-1.002
Prenatal infection	0.716	0.333	1	2.046	2.95-1.065
Family history	0.481	0.259	1	0.975	1.234-1.372
Abortion history	0.026	0.267	1	0.233	0.577-0.135
Abnormalities detected in scanning	0.629	0.304	1	0. 618	1.00-0.294
Maternal age at 20-25	0.165	0.319	0	0.0512	0.075-0.0216
Maternal age at 25-30	0.243	0.357	1	0.384	0.566-0.190
Maternal age at 30-35	0.114	0.53	1	1.107	1.78-0.016
Maternal age at 35-40	0.178	0.416	1	1.163	2.67-0.070
Paternal age at 20-25	0.136	0.404	0	0.028	0.089-0.014
Paternal age at 25-30	0.58	0.261	1	0.236	0.558-0.316
Paternal age at 30-35	0.182	0.35	1	0.821	1.19-0.101
Paternal age at 35-40	0.214	0.275	1	1.893	2.189-0.723
Order of child birth = 1st	0.234	0.302	1	1.298	1.546-0.118
Order of child birth = 2nd	0.243	0.357	1	0.791	0.996-0.438
Order of child birth = 3rd	0.019	0.832	1	0.784	1.08-0.390
Order of child birth = 4th	0.059	0.954	0	0.045	0.543-0.225
Order of child birth = 5th	0.43	1.65	1	0.067	0.441-0.023
Lower SES	0.77	0.509	0	1.836	2.57-1.679
Lower middle SES	0.026	0.267	1	0.975	1.324-0.577
Middle SES	0.178	0.416	1	0.837	1.99-0.370
Upper middle SES	0.124	0.225	1	0.567	0.999-0.723
Upper SES	0.981	0.287	1	0.0747	0.222-0.021

**Table 18:** Multivariate logistic regression analysis for CP The strength of association for cleft palate (CP) was assessed using logistic regression and expressed as odds ratios (exponential β) with a 95% CI upper and lower limits. Consanguinity showed a 2.390-fold increased risk for CP, with a 95% CI values ranging from 2.222 to 3.321. Lack of prenatal folic acid use showed a risk of 1.001, with a 95% CI bounds of 0.463-1.00. Prenatal infections had a risk value of 0.563 for CP, with upper and lower 95% CI limits of 1.73-0.370. Family history was associated with a 1.238-fold increased risk, with a 95% CI values of 0.723-1.987. Previous abortion history showed a risk of 0.191, with a 95% CI boundaries of 0.06-0.438. Abnormalities detected in scans showed a 1.836-fold increased risk for CP at 95% CI. Maternal age groups of 20-25, 25-30, 30-35, and 35-40 years showed risks of 0.0113, 0.216, 1.345, and 1.017, with corresponding 95% CI values of 0.011-0.055, 0.190-2.79, 0.167-2.0, and 1.009-2.49. Likewise, paternal age in the same groups showed risks of 0.032, 0.821, 0.236, and 1.893, with a 95% CI limits of 0.022-0.789, 0.316-1.073, 0.101-0.404, and 1.723-1.944 for CP. For birth order (1st-5th), the risks were 2.298, 0.784, 0.891, 0.321, and 0.225, respectively, with a 95% CI boundaries of 1.418-3.123, 0.390-1.186, 0.438-1.119, 0.537-0.735, and 0.345-0.414. Socioeconomic status showed risks of 1.985 for the lower class, 0.738 for the lower middle class, 0.375 for the middle class, 0.237 for the upper middle class, and 0.193 for the upper class, with a 95% CI values of 1.612-2.123, 0.799-1.00, 0.577-0.737, 0.370-0.564, and 0.222-0.518, respectively, for CP.

Intercept	β	Std. error	df	Odds ratio	95% confidence interval for odds ratio
Consanguinity	0.941	0.287	1	2.39	3.321-2.222
Prenatal folic acid usage	0.591	0.393	1	1.001	1.00-0.463
Prenatal infection	0.178	0.416	1	0.563	1.73-0.370
Family history	0.214	0.275	1	1.238	1.987-0.723
Abortion history	0.234	0.302	1	0.191	0.438-0.06
Abnormalities detected in scans	0.608	0.261	1	1.836	2.432-1.101
Maternal age at 20-25	0.119	0.186	0	0.0113	0.055-0.011
Maternal age at 25-30	0.143	0.287	1	0.216	0.279-0.190
Maternal age at 30-35	0.141	0.53	1	1.345	2.00-0.167
Maternal age at 35-40	0.187	0.416	1	1.017	2.49-1.009
Paternal age at 20-25	0.347	0.231	0	0.032	0.789-0.022
Paternal age at 25-30	0.128	0.35	1	0.821	1.073-0.316
Paternal age at 30-35	0.68	0.261	1	0.236	0.404-0.101
Paternal age at 35-40	0.241	0.275	1	1.893	1.944-1.723
Order of child birth = 1st	0.208	0.357	1	2.298	3.123-1.418
Order of child birth = 2nd	0.091	0.832	1	0.784	1.186-0.390
Order of child birth = 3rd	0.243	0.302	1	0.891	1.119-0.438
Order of child birth = 4th	0.454	0.0792	0	0.321	0.735-0.537
Order of child birth = 5th	0.288	0.468	1	0.225	0.414-0.345
Lower SES	0.765	0.549	0	1.985	2.123-1.612
Lower middle SES	0.124	0.225	1	0.738	1.000.0.799
Middle SES	0.026	0.267	1	0.375	0.737-0.577
Upper middle SES	0.178	0.416	1	0.237	0.564-0.370
Upper SES	0.981	0.287	1	0.193	0.518-0.222

**Table 19:** Multivariate logistic regression analysis for CLP Multiple logistic regression was performed to evaluate the association between risk factors and cleft lip and palate (CLP). The results are presented as odds ratios (exponential β) with their corresponding 95% confidence intervals. For CLP, the risk associated with consanguinity was 1.640 times, with a 95% CI of 2.12-1.22. Lack of prenatal folic acid use showed a 1.313-fold risk for CLP, with a 95% CI of 1.414-0.463. Prenatal infection showed a lower risk of 0.777 times (95% CI: 0.875-0.370). Family history showed a risk of 0.867 times, with a 95% CI of 1.110-0.723. Previous abortion history showed a risk of 0.159 times with a 95% CI of 0.438-0.130. Abnormalities detected in scans had a 2.180-fold higher risk with a 95% CI of 2.41-1.101. Maternal age groups of 20-25, 25-30, 30-35, and 35-40 years showed risks of 0.012, 0.116, 2.154, and 1.198, respectively, with 95% CIs of 0.059-0.010, 0.336-0.190, 3.176-2.076, and 1.489-1.079. Paternal age within the same groups showed risks of 0.030, 0.298, 0.981, and 1.893 with corresponding 95% CIs of 0.147-0.008, 0.755-0.316, 1.01-0.701, and 2.05-1.723. Birth order (1st-5th) showed risks of 1.298, 0.784, 0.791, 0.055, and 0.237 times for CLP, with 95% CIs of 2.070-1.13, 0.976-0.390, 0.954-0.438, 0.987-0.099, and 0.376-0.114. Socioeconomic status (lower, lower middle, middle, upper middle, upper) showed risks of 2.79, 0.864, 0.575, 0.238, and 0.088, with 95% CIs of 2.87-1.72, 1.212-0.543, 0.864-0.577, 0.333-0.227, and 0.234-0.034, respectively.

Intercept	β	Std. Error	df	Odds ratio	95% confidence interval for odds ratio
Consanguinity	0.43	0.186	1	1.64	2.22-1.22
Prenatal history of folic acid	0.177	0.457	1	1.313	1.414.0.463
Prenatal infection	0.653	0.318	1	0.777	0.875-0.370
Family history	0.346	0.277	1	0.867	1.110-0.723
Abortion history	0.522	0.408	1	0.159	0.438-0.130
Abnormalities detected in scans	0.617	0.144	1	2.18	2.41-1.101
Maternal age at 20-25	0.124	0.386	0	0.012	0.059-0.010
Maternal age at 25-30	0.173	0.207	1	0.116	0.336-0.190
Maternal age at 30-35	0.141	0.53	1	2.154	3.176-2.076
Maternal age at 35-40	0.119	0.517	1	1.198	1.489-1.079
Paternal age at 20-25	0.134	0.567	0	0.03	0.147-0.008
Paternal age at 25-30	0.128	0.35	1	0.298	0.755-0.316
Paternal age at 30-35	0.68	0.261	1	0.981	1.01-0.701
Paternal age at 35-40	0.241	0.275	1	1.893	2.05-1.723
Order of child birth = 1st	0.208	0.357	1	1.298	2.070.1.13
Order of child birth = 2nd	0.091	0.832	1	0.784	0.976-0.390
Order of child birth = 3rd	0.243	0.302	1	0.791	0.954-0.438
Order of child birth = 4th	0.843	0.578	0	0.055	0.987-0.099
Order of child birth = 5th	0.406	0.144	0	0.237	0.376-0.114
Lower SES	0.999	0.875	0	2.79	2.87-1.72
Lower middle SES	0.543	0.212	1	0.864	1.212-0.543
Middle SES	0.036	0.149	1	0.575	0.864-0.577
Upper middle SES	0.123	0.517	1	0.238	0.333-0.227
Upper SES	0.978	0.386	1	0.088	0.234-0.034

## Discussion

Congenital disabilities are among the leading causes of child morbidity. A 2001 study by Venkatesh [13] in three districts of India reported that clefts occurred in 1.09 per 1000 births, with a male predominance that was statistically significant (p < 0.05). Similarly, Mossey and Little [14] studied 150 patients with CL and CP and found a higher prevalence in male patients, with high statistical significance (p < 0.001), consistent with the findings of our study.

In a retrospective study by Sakran et al. conducted from 2017 to 2019 involving 1,260 patients, 13 factors were identified as significantly associated with CL and CP: male gender, advanced paternal and maternal age at birth, lack of folic acid supplementation, consanguinity, birth order in multiple births, positive family history, prenatal infections, history of abortion, maternal smoking, low maternal childbearing age, malnutrition, and abnormalities detected on prenatal scans [15]. In the present study, we also considered factors such as age, gender, complete CL by gender, incomplete CL by gender, alveolar involvement in CL, consanguinity, paternal and maternal age, birth order, maternal folic acid supplementation, family history, previous abortions, abnormalities detected on scans, and socioeconomic status.

In our study, among patients with complete CL, 70 had unilateral clefts and 28 had bilateral clefts. Of these, 14 were right-sided and 56 were left-sided, with a statistically significant association (p = 0.01). This indicates that clefts were more common on the left side and equally distributed between genders, although bilateral clefts were more frequent in males. For incomplete CL, six males had unilateral clefts and six had bilateral clefts. Among females, there were no cases of bilateral CL, and 11 had unilateral clefts (right side: 11; left side: 6). The association was not statistically significant (p = 0.17). Regarding CL involving the alveolus, 27 patients were affected (17 males and 10 females), with 9 right-sided, 15 left-sided, and 3 bilateral clefts. The results were not statistically significant for left-sided alveolar involvement (p = 0.23). These findings are consistent with a systematic review by Gundlach and Maus [16], which reported that complete clefts are more common on the left side and occur more frequently in males, while involvement of the primary and secondary palate was more common in females (p = 0.01).

In a descriptive hospital-based study conducted by Aleman and Martinez across four hospitals, researchers compared 144 nonsyndromic cleft cases with 144 healthy controls [17]. They found consanguinity to be a major contributing factor (p < 0.001). These findings are consistent with the current study, in which 131 of 404 patients had a positive history of consanguinity (p ≤ 0.001). Similarly, Bille et al. [18] analyzed 1,489,014 births in Denmark between 1973 and 1996 and found a significant association between CLP and advanced paternal age (p = 0.001). The highest percentage of cases occurred in fathers aged 36-40 years (23,789 patients), followed by 31-35 years (89 patients) and 20-30 years (78 patients), with statistical significance (p = 0.01). This is comparable to our study, in which 237 patients were in the 36-40-year paternal age group and 89 patients in the 31-35-year group, with a statistically significant p-value of 0.01, indicating increased prevalence of OFCs with advancing paternal age.

Bille et al. [18] also reported that the mean maternal age was 39 years. Among 110 patients with maternal ages ranging from 18 to 60 years, CL and CP were more common in mothers over 35 years of age. Similarly, de Souza Maurique et al. [19], in a study of ICU patients from 2012 to 2018, found a higher incidence of CL and CP associated with advanced maternal age (p < 0.05), with a 35.2% increase in risk for each year of maternal age above 40. In our study, the highest incidences of CL and CP were observed in maternal age groups 36-40 years (94 patients), 31-35 years (224 patients), and 20-30 years (83 patients), with a statistically significant association (p ≤ 0.001), indicating a strong link between higher maternal age and clefts.

Jac-Okereke and Onah [20] conducted a descriptive examination of birth records in Nigeria from 2007 to 2011, analyzing 139 males and 123 females, and found that firstborn children were at greater risk of clefts (p < 0.001). Likewise, a systematic review by Vieira [10] highlighted the crucial role of gene-gene and gene-environment interactions in determining OFCs. In our study, 283 of 404 affected children were firstborn, and we observed that the risk of clefts decreased with increasing birth order, with firstborn status showing a statistically significant association (p = 0.01).

Kelly et al. [21] investigated the association between preconception folic acid use and the prevalence of CL and CP, reporting a significantly higher incidence of clefts in mothers who did not take folic acid during gestation (p < 0.001). In our study, based on detailed case histories, 342 mothers reported no folic acid usage, while 62 mothers had a history of supplementation. This association was highly significant (p ≤ 0.001), highlighting the importance of folic acid in preventing CL and CP.

Shi et al. [22], in a systematic review, examined the relationship between maternal infections and CL and CP, finding no association between serum-specific antibodies for influenza, rubella, CMV, EBV, parotitis, or HBsAg and clefts, despite these infections being common during pregnancy. In our study, 346 of 404 mothers (85.6%) had a history of prenatal infections, while 58 had no such history. The association was highly significant (p ≤ 0.001), suggesting that prenatal infections may contribute to the development of CL and CP.

In our study, 300 patients had a positive family history of CL and CP, while 104 had no family history. This association was statistically significant (p = 0.01). These results are consistent with González et al. [23], who examined 835 patients (504 boys and 331 girls) and reported a highly significant association between family history and cleft formation (p < 0.001), highlighting the role of inherited genes. Regarding abortion history, 100 patients in our study had a previous history of abortion, while the remaining 304 did not. There was no statistically significant association with CL and CP (p = 0.093), indicating no correlation. This finding aligns with de Moraes Pereira et al. [24], who analyzed 1,004 patients (502 with a history of abortion and 502 without) and found no significant difference in the occurrence of nonsyndromic clefts (p < 0.07).

In our study, among 404 patients, 265 had a history of abnormalities detected during prenatal growth scans, while 139 patients had no such abnormalities. This association was highly significant (p ≤ 0.001). Similarly, Christ et al. [25] studied 200 prenatal scans and found a strong correlation between cleft detection via ultrasound and the diagnosis of clefts before birth (p ≤ 0.001). These patients were referred for consultation with plastic surgeons immediately after birth, allowing timely intervention and corrective procedures.

In a systematic review, Vyas et al. [26] highlighted that successful management of cleft patients requires a multidisciplinary approach, involving specialties such as oral and maxillofacial surgery, otorhinolaryngology, genetics, speech therapy, orthodontics, prosthodontics, and psychiatry. Early detection was emphasized as essential to save time and prevent complications. Similarly, Vu et al. [27], in a study of 4,164 individuals using multiple logistic regression analysis, found that socioeconomic status plays a crucial role in the prevalence of CL and CP (p < 0.005), largely due to poor literacy and limited access to primary education. In our study, 344 patients belonged to lower and lower-middle-class groups, 51 to middle and upper-middle-class groups, and 9 to the upper class. The association was highly significant (p ≤ 0.001), highlighting the influence of socioeconomic factors, nutrition, and pre- and postnatal maternal care on the occurrence of clefts.

In a study by Mendonca [28] involving 318 individuals with nonsyndromic clefts, it was found that lack of prenatal folic acid supplementation increased the risk of cleft deformities (OR = 0.62). Similarly, in our study, multivariate logistic regression analysis identified several significant risk factors for CL. Lack of prenatal folic acid supplementation had an OR of 1.89, consanguinity had an OR of 2.113, and prenatal infections had an OR of 2.046. Advanced paternal age (35-40 years) was associated with an OR of 1.893. Lower socioeconomic status had an OR of 1.836, first-born status had an OR of 1.298, and maternal age 35-40 years and 30-35 years were associated with ORs of 1.163 and 1.107, respectively. These findings indicate that multiple parental, prenatal, and socioeconomic factors significantly contribute to the risk of CL.

In a systematic review and meta-analysis by Ács et al. [29], which included 5,830 studies, maternal obesity was associated with an increased risk of clefts (OR = 1.28), maternal underweight (OR = 1.21), type 1 diabetes (OR = 1.75), and both prenatal maternal hypertension and active smoking during pregnancy (OR = 1.55 each). In the current study, multiple logistic regression analysis revealed several factors significantly increasing the risk of CP. Children of consanguineous parents had an OR of 2.390. First-born status was associated with an OR of 2.298. Lower socioeconomic status showed an increased risk (OR = 1.985), while advanced paternal age (35-40 years) had an OR of 1.893. Abnormalities detected on prenatal ultrasound were associated with an OR of 1.836. Maternal age 30-35 years had an OR of 1.345, and 35-40 years had an OR of 1.017. Positive family history of CP carried an OR of 1.23, and lack of prenatal folic acid supplementation had an OR of 1.01. Collectively, these confounding factors substantially increase the risk of CP.

The current study identified several factors significantly associated with CLP. Lower socioeconomic status was associated with an elevated risk (OR = 2.79), while abnormalities detected on prenatal ultrasound had an OR of 2.18. Advanced paternal age (35-40 years) showed an OR of 1.893. Maternal age 30-35 years had an OR of 2.154, and 35-40 years had an OR of 1.198. Consanguinity was associated with an OR of 1.64, lack of prenatal folic acid supplementation with an OR of 1.313, and first-born status with an OR of 1.298, as determined by multivariate logistic regression analysis. Similarly, a prospective cross-sectional study by Zhou et al. [30] on 143,118 infants, including 2,984 with birth defects, found that first-born status in multiple births (OR = 1.44), advanced paternal age > 40 years (OR = 2.20), and 30-35 years (OR = 1.16), as well as congenital metabolic disorders in males (OR = 3.86) and females (OR = 2.35), were significantly associated with the risk of OFCs.

Limitations of the study

This study does not establish a direct cause-and-effect relationship due to its observational design. A major limitation is that data were collected from a single tertiary care hospital, which may limit the generalizability of the findings. Including data from multiple hospitals across diverse regions could capture additional factors influencing orofacial clefts. Furthermore, incorporating information on postoperative follow-up and care could enhance the depth and applicability of the study.

Future scope

CL, CP, and CLP are among the most common congenital anomalies and can significantly impact an individual’s health, well-being, and psychological development. Prevention of these conditions should be prioritized over treatment. Longitudinal studies focusing on postoperative care, feeding difficulties, psychological outcomes, and the effectiveness of various surgical approaches are essential to reduce long-term morbidity in patients with clefts.

## Conclusions

The current study identifies male gender as a significant risk factor for cleft formation, whereas incomplete CL and CL with alveolar involvement showed no significant association with gender. Other important risk factors for CL, CP, and CLP include genetic, environmental, and sociodemographic factors, such as consanguinity, advanced parental age, inadequate maternal folic acid intake during gestation, first-born status, and low socioeconomic status. Maternal infections were more strongly associated with CL, while positive family history and abnormalities detected on prenatal scans were more strongly associated with CP and CLP. Previous history of abortions showed no significant association with clefts. These findings highlight the importance of preventive strategies, including genetic counseling and improved maternal nutrition. Early detection through prenatal diagnostics is crucial to reducing the incidence of OFCs and optimizing patient outcomes.

## Appendices

Supplemental material 1

**Table 20:** Case history proforma of the study

Parameter	Response/details
Age in months:	
Sex:	
Complete cleft lip:	Yes/No
Incomplete cleft lip:	Yes/No
Cleft lip with involvement of the alveolus:	Yes/No
Marital status:	Yes/No
Consanguinity:	Consanguineous/Non-consanguineous
Paternal age at birth:	Mentioned in years
Maternal age at birth:	Mentioned in years
Order of child birth:	1st/2nd/3rd/4th/5th
Prenatal intake of folic acid:	Yes/No
If yes, weeks of gestation:	
Prenatal infection:	Yes/No
If yes, type of infection and duration:	
Prenatal scanning done:	Yes/No
If yes, abnormality detected and period:	
Family history of clefts:	Yes/No
Previous abortions:	Yes/No
If yes, number:	
Feeding modalities given:	
Feeding difficulties:	Yes/No
Socioeconomic status:	Lower/Lower middle/Middle/Upper middle/Upper
Any other systemic illness of the child:	Yes/No
If yes, type of illness:	

Notes

There are a total of 16 tables and 15 graphical figures that present the data from our study and provide clear visual representations. However, the journal guidelines allow only 25 media items in total. In compliance with these requirements, we are unable to upload the graphical figures. We respectfully hope for your understanding.

## References

[1] Reddy SG, Reddy RR, Bronkhorst EM, Prasad R, Ettema AM, Sailer HF, Bergé SJ (2010). Incidence of cleft lip and palate in the state of Andhra Pradesh, South India. Indian J Plast Surg.

[2] Mossey PA, Little J, Munger RG, Dixon MJ, Shaw WC (2009). Cleft lip and palate. Lancet.

[3] Som PM, Naidich TP (2013). Illustrated review of the embryology and development of the facial region, part 1: early face and lateral nasal cavities. AJNR Am J Neuroradiol.

[4] Gorlin RJ, Cohen MM, Hennekam RC (2001). Syndromes of the Head and Neck, 4th Edition. Oxford University Press.

[5] Allam E, Windsor LJ, Stone C (2014). Cleft lip and palate: etiology, epidemiology, preventive and intervention strategies. Anat Physiol.

[6] Kasten EF, Schmidt SP, Zickler CF (2008). Team care of the patient with cleft lip and palate. Curr Probl Pediatr Adolesc Health Care.

[7] Jamilian A, Sarkarat F, Jafari M, Neshandar M, Amini E, Khosravi S, Ghassemi A (2017). Family history and risk factors for cleft lip and palate patients and their associated anomalies. Stomatologija.

[8] Elahi MM, Jackson IT, Elahi O, Khan AH, Mubarak F, Tariq GB, Mitra A (2004). Epidemiology of cleft lip and cleft palate in Pakistan. Plast Reconstr Surg.

[9] Lei RL, Chen HS, Huang BY (2013). Population-based study of birth prevalence and factors associated with cleft lip and/or palate in Taiwan 2002-2009. PLoS One.

[10] Vieira AR (2008). Unraveling human cleft lip and palate research. J Dent Res.

[11] Sharma R (2017). Revised Kuppuswamy's socioeconomic status scale: explained and updated. Indian Pediatr.

[12] Kernahan DA, Stark RB (1958). A new classification for cleft lip and cleft palate. Plast Reconstr Surg Transplant Bull.

[13] Venkatesh R (2009). Syndromes and anomalies associated with cleft. Indian J Plast Surg.

[14] Mossey P, Little J (2009). Addressing the challenges of cleft lip and palate research in India. Indian J Plast Surg.

[15] Sakran KA, Abotaleb BM, Al-Rokhami RK (2022). Analysis of environmental exposures for nonsyndromic cleft lip and/or palate: a case-control study. Iran J Public Health.

[16] Gundlach KK, Maus C (2006). Epidemiological studies on the frequency of clefts in Europe and world-wide. J Craniomaxillofac Surg.

[17] Aleman RM, Martinez MG (2017). Tessier 3 cleft in a pre-Hispanic anthropomorphic figurine in El Salvador, Central America. Cleft Palate Craniofac J.

[18] Bille C, Knudsen LB, Christensen K (2005). Changing lifestyles and oral clefts occurrence in Denmark. Cleft Palate Craniofac J.

[19] de Souza Maurique L, Muniz FW, Silveira NP, Camassola M, de Oliveira BM (2022). Higher maternal age is associated with higher occurrence of cleft lip/palate in neonates under intensive care. Braz J Oral Sci.

[20] Jac-Okereke CA, Onah II (2017). Epidemiologic indices of cleft lip and palate as seen among Igbos in Enugu, Southeastern Nigeria. JCLPCA.

[21] Kelly D, O'Dowd T, Reulbach U (2012). Use of folic acid supplements and risk of cleft lip and palate in infants: a population-based cohort study. Br J Gen Pract.

[22] Shi FP, Huang YY, Dai QQ, Chen YL, Jiang HY, Liang SY (2023). Maternal common cold or fever during pregnancy and the risk of orofacial clefts in the offspring: a systematic review and meta-analysis. Cleft Palate Craniofac J.

[23] González BS, López ML, Rico MA, Garduño F (2008). Oral clefts: a retrospective study of prevalence and predisposal factors in the State of Mexico. J Oral Sci.

[24] de Moraes Pereira MC, Silva CM, de Queiroz TB, Neves LT (2023). Oral cleft and maternal history of spontaneous abortion: a case-control study. Cleft Palate Craniofac J.

[25] Christ JE, Meininger MG (1981). Ultrasound diagnosis of cleft lip and cleft palate before birth. Plast Reconstr Surg.

[26] Vyas T, Gupta P, Kumar S, Gupta R, Gupta T, Singh HP (2020). Cleft of lip and palate. A review. J Family Med Prim Care.

[27] Vu GH, Warden C, Zimmerman CE (2022). Poverty and risk of cleft lip and palate: an analysis of United States birth data. Plast Reconstr Surg.

[28] Mendonca VJ (2019). Maternal folic acid intake and risk of nonsyndromic orofacial clefts: a hospital-based case-control study in Bangalore, India. Cleft Palate Craniofac J.

[29] Ács M, Cavalcante BG, Bănărescu M (2024). Maternal factors increase risk of orofacial cleft: a meta-analysis. Sci Rep.

[30] Zhou X, He J, Wang A, Hua X, Li T, Shu C, Fang J (2024). Multivariate logistic regression analysis of risk factors for birth defects: a study from population-based surveillance data. BMC Public Health.

